# Change in perioperative neutrophil-lymphocyte ratio as a potential predictive biomarker for chronic postsurgical pain and quality of life: an ambispective observational cohort study

**DOI:** 10.3389/fimmu.2023.1177285

**Published:** 2023-04-12

**Authors:** Bin Shu, Fang Xu, Xuemei Zheng, Yamei Zhang, Qi Liu, Shiqi Li, Jie Chen, Yuanjing Chen, He Huang, Guangyou Duan

**Affiliations:** Department of Anesthesiology, The Second Affiliated Hospital, Chongqing Medical University, Chongqing, China

**Keywords:** chronic postsurgical pain, inflammation, neutrophil, lymphocyte, NLR, risk factor

## Abstract

**Introduction:**

Accurate and accessible predictors of chronic postsurgical pain (CPSP) to identify high-risk postsurgical patients are prerequisite for preventive and interventional strategies. We investigated the incidence and risk factors of CPSP after abdominal surgery, with a focus on plasma immunological markers.

**Materials and methods:**

This was a retrospective analysis of patients who underwent abdominal surgery under general anesthesia at a tertiary center between January 2021 and January 2022. The preoperative demographics, laboratory test data, and surgical factors of the participants were collected from the electronic medical record system. Postoperative pain intensity and living conditions at 1 year after discharge from the hospital were assessed *via* a phone survey. Univariate and multivariate analyses were used to explore independent risk factors associated with CPSP.

**Results:**

A total of 968 patients were included, and 13.53% (n = 131 of 968) of patients reported CPSP 1 year after surgery. Patients with older age, open surgery, higher American Association of Anesthesiologists classification, patient-controlled intravenous analgesia application, longer surgery duration, higher postoperative absolute neutrophil count, and neutrophil-lymphocyte ratio (NLR), lower postoperative absolute lymphocyte count, and higher white blood cell count, were more likely to suffer from CPSP. A changed ratio of NLR (postoperative to preoperative) ≥ 5 significantly correlated with CPSP, moderate to severe pain, maximum numeric rating score since discharge from the hospital, and affected quality of life.

**Discussion:**

The changed ratio of NLR could be used for the early identification of patients at risk for CPSP and affect the quality of life to alert the clinician to undertake further assessment.

## 1 Introduction

Chronic postsurgical pain (CPSP) is defined as chronic pain that develops or increases in intensity after a surgical procedure and persists beyond the healing process after surgery, excluding other known causes (1). It occurs in up to 85% of patients with amputation, 30–50% after breast surgery, and approximately 10% after inguinal hernia repairs (2), which means that more than 32 million additional people globally suffer from long-lasting pain every year (3). Owing to discomfort, distress, disability, increased medical burden, and opioid abuse, CPSP has become a major public health problem (4–6). It is anticipated that identifying the potential early predictive factors of CPSP would be helpful for understanding and exploring the underlying mechanisms and developing better therapeutic strategies.

The causes of CPSP are not fully known; however, several risk factors, including patient- and treatment-related factors, have been established. The main predictive patient-related risk factors including demographic factors (younger adults, female sex, single or living alone, and smoking), medical factors (more medical comorbidities, higher body mass index (BMI), with or with greater presurgical pain), and psychosocial factors (higher levels of presurgical anxiety, depression, and pain catastrophizing). Among treatment-related risk factors, longer duration of surgery, higher surgical risk of nerve damage, more surgical complications, and poorly controlled acute postoperative pain have been suggested as important risk factors (4, 7–15). These risk factors are interrelated and contribute to the development and maintenance of CPSP (16). Accurate prediction of CPSP is a pressing prerequisite for CPSP prevention and intervention. Some studies have proposed predictive models for CPSP (5, 13, 17–20), but too many variables and their subjective nature make them less operable in practice.

Inflammation plays an important role in acute pain (21), but it has been less studied in chronic pain. Ongoing inflammation plays an important role in mediating the transition from acute postoperative pain to chronic pain (22). The pain- and inflammation-induced release of pro-cytokines and chemokines is responsible for peripheral and central sensitization, which are important mechanisms of CPSP (12). The direct effect of perioperative changes in inflammatory status on the development of postoperative chronic pain remains unclear and needs to be elucidated. Thus, it has been hypothesized that increased inflammatory factors and indicators of the preoperative inflammatory response may contribute to CPSP.

This ambispective observational cohort study aimed to retrospectively analyze a comprehensive list of risk factors for CPSP in patients who have undergone abdominal surgery in the last few years at a tertiary center. To generate a basis for preventive and treatment strategies, the focus was on known preoperative patient status, plasma immunological indicators, treatment characteristics, and how CPSP was predicted.

## 2 Materials and methods

### 2.1 Study population

We conducted an ambispective observational cohort study of patients undergoing abdominal surgery treated at the Second Affiliated Hospital of Chongqing Medical University in the Republic of China between January 2021 and January 2022. Patients who met the following inclusion criteria were enrolled: patients who underwent elective abdominal endoscopic or open surgery under general anesthesia; American Society of Anesthesiologists (ASA) physical status II–III; and between 18 and 70 years of age. Patients were excluded if they met any of the following criteria: death at the time of follow-up, inability to communicate, state of emergency before surgery, and lack of data needed for laboratory tests or follow-up to assess inflammatory status.

This study was approved by the Medical Ethics Committee of the Second Affiliated Hospital of Chongqing Medical University (Approval Document No.2023-18-1). The study was conducted in accordance with the Declaration of Helsinki, and the confidentiality of patient data was guaranteed. Because the study was based on retrospective data and telephone follow-ups, oral informed consent was obtained. Unidentified data supporting the conclusion of this article can be acquired on reasonable request without any reservation through email to the corresponding author.

### 2.2 Data collection

Patient data for this study were collected from an electronic medical record system. The collected data included demographics such as sex, age, height, weight, BMI, ASA grading, and laboratory test data, including inflammation-related indicators such as white blood cell (WBC) count, absolute neutrophil count (ANC), absolute lymphocyte count (ALC), and neutrophil-to-lymphocyte ratio (NLR). In this study, all preoperative examinations and test results were collected within three days prior to the operation, and those with multiple preoperative examinations were included in the results on the nearest operation day. All postoperative plasma inflammatory blood cell parameters were collected within one day of the operation.

WBC count, ANC, and ALC are widely known to reflect immune function and inflammatory state in the body (23, 24). The NLR is the ratio of the neutrophil count to the lymphocyte count in routine blood tests, which can comprehensively reflect the increase in neutrophils and decrease in lymphocytopenia in the inflammatory state (25). Other data included the surgical approach, including endoscopic and open surgery, type of surgery including gastrointestinal, hepatobiliary, or gynecological surgery, transversus abdominis plane block (TAP) type including unilateral and bilateral, whether additional analgesia was needed, whether patient-controlled intravenous analgesia (PCIA) was used, maximum incisional numeric rating scale (NRS) score at 24 hours postoperation, and surgery duration.

### 2.3 Outcome measures

The primary outcome of interest was CPSP, defined as pain complain or NRS score higher than 0 since three months post-operation at the point of telephone interview (11–13 months after surgery).

Secondary outcomes included the maximum NRS score after hospital discharge, moderate to severe pain (pain with an NRS score of 4 or higher), and whether CPSP affected sleeping, daily life, or work.

### 2.4 Statistical analysis

All normally distributed continuous data were summarized as the mean (standard deviation), non-normally distributed continuous data were presented as median (25th–75th percentile), and quantity (percentage) was used to represent qualitative variables. A paired sample t-test was used to compare groups of normally distributed data, and the Wilcoxon rank-sum test was used for non-normally distributed data. The Chi-square test was used for comparison between groups, and Fisher’s exact probability method was used when the theoretical frequency was less than five. Repeated measures ANOVA or Friedman’s test was used to compare the changes in more than two levels. Bonferroni correction was used to adjust the *P* values for multiple comparisons. All statistical analyses were performed using SPSS software (IBM SPSS Statistics for Windows, Version 26.0. Armonk, NY: IBM Corp.). A *P* value less than 0.05 was considered statistically significant.

First, patients were grouped according to the occurrence of CPSP, and then different groups were compared. Because all inflammatory indicators were abnormally distributed, the Mann–Whitney U test was used for comparison. Second, the receiver operating characteristic curve was used to explore the predictive abilities of these different inflammatory indicators in distinguishing the early postoperative outcomes of patients undergoing abdominal surgery, and the optimal cutoff point was calculated based on the Youden index score. Finally, the optimal cutoff value was used to group the patients according to the level of inflammation, and univariate logistic regression was used to explore the predictive efficacy of different preoperative factors for major clinical outcomes. Factors with *P* < 0.05 were included in the multivariate logistic regression to further verify the predictive efficacy of the inflammation index for postoperative outcomes.

## 3 Results

### 3.1 Baseline characteristics of subjects

**Figure 1:**
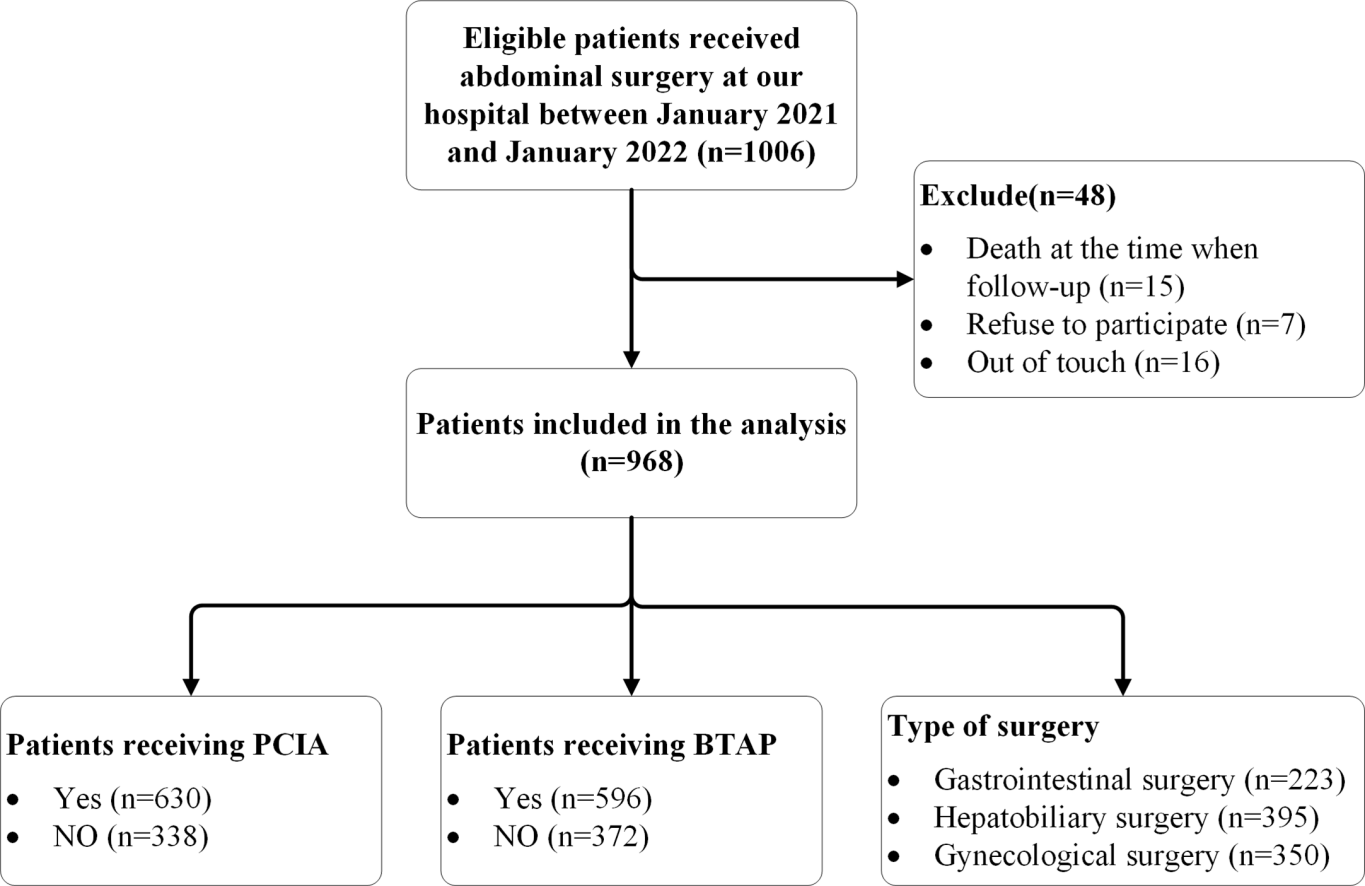
Flow diagram representing patient enrollment. PCIA indicates Patient Controlled Intravenous Analgesia; BTAP indicates bilateral transversus abdominis plane block.

This study included 1,006 patients. Fifteen patients died at the time of follow-up, 16 were out of touch, 7 refused to participate; therefore, 968 eligible cases were included in the statistical analysis. Among these 968 patients, 630 received PCIA and 596 received bilateral TAP (BTAP). A total of 223 gastrointestinal surgeries, 395 hepatobiliary surgeries, and 350 gynecological surgeries were performed (**Figure 1**). The incidence of CPSP was 13.53% (n = 131 of 968). The mean age of the total cohort was 48 years, and 71.1% of the patients were female. Endoscopic surgery was performed in 90% of the patients, with an average surgery duration of 134.57 min. BTAP was performed in 61.6% of the patients, while the remaining 38.4% underwent unilateral TAP. PCIA was administered to 65.1% of the patients. The maximum incisional NRS score at 24 h was 3.43, and 30.8% of patients required additional analgesia. The demographic and baseline data of all subjects are shown in **Table 1**.

**Table 1:** Participants’ characteristics.

Characteristics		n=968
Age	Year, mean (SD)	48 ± 13
Sex	Male, n (%)	280 (28.9%)
	Female, n (%)	688 (71.1%)
Height	cm, mean (SD)	160.39 ± 7.97
Weight	kg, mean (SD)	61.23 ± 10.84
BMI	kg/m^2^, mean (SD)	23.84 ± 5.08
Surgical approach	Endoscope, n (%)	871 (90.0%)
	Open, n (%)	97 (10.0%)
ASA	II, n (%)	549 (56.7%)
	III, n (%)	419 (43.3%)
Maximum incisional NRS score at 24h	mean (SD)	3.43 ± 1.39
Additional Analgesia	Yes, n (%)	298 (30.8%)
	No, n (%)	670 (69.2%)
PCIA	Yes, n (%)	630 (65.1%)
	No, n (%)	338 (34.9%)
TAP	Unilateral, n (%)	372 (38.4%)
	Bilateral, n (%)	596 (61.6%)
Type of Surgery	Gastrointestinal, n (%)	223 (23.0%)
	Hepatobiliary, n (%)	395 (40.8%)
	Gynecological, n (%)	350 (36.2%)
Surgery duration	Min, mean (SD)	134.57 ± 90.61

CPSP indicates chronic postsurgical pain; BMI indicates body max index; ASA indicates American Society of Anesthesiologists physical status; NRS indicates numeric rating scale; PCIA indicates Patient Controlled Intravenous Analgesia; TAP indicates transversus abdominis plane block.

### 3.2 Plasma inflammatory blood cell parameters are strongly associated with a high risk of CPSP

**Figure 2:**
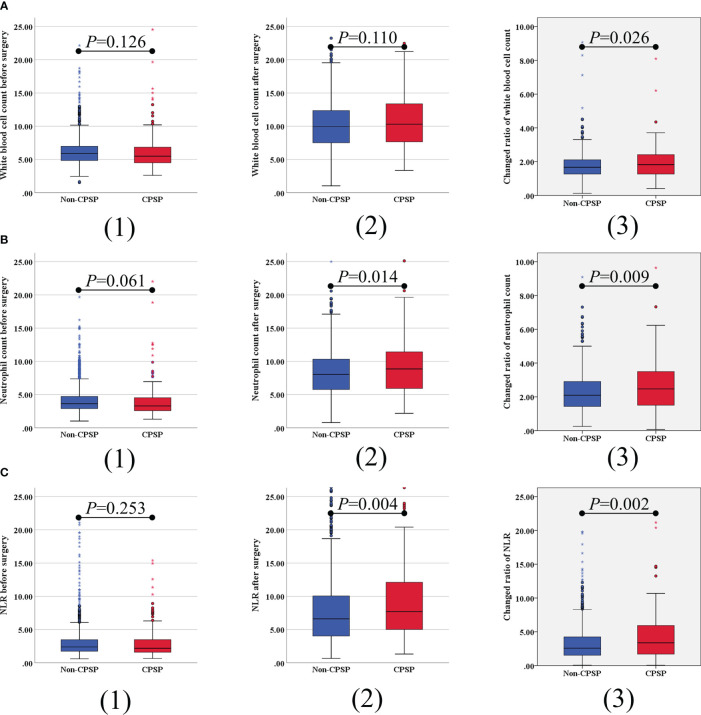
Univariate analysis of correlation between plasma inflammatory blood cell parameters and CPSP. The preoperative, postoperative and change ratio of white blood cell count **(A)**, absolute neutrophil count **(B)**, and neutrophil-lymphocyte ratio (NLR) **(C)** were compared. Error bars indicate 95% CIs.

The sociodemographic and medical history of the subjects compared with or without CPSP are presented in **Table 2**. The results of the univariate analysis showed that patients in the CPSP and non-CPSP groups did not differ strikingly in sex, height, weight, BMI, maximum incisional NRS score at 24 h, whether additional analgesia was needed, nerve block type, type of surgery, Preoperative WBC count, Preoperative ANC, Preoperative ALC, Preoperative NLR, or Postoperative WBC count. The factors associated with CPSP included older age (*P* = 0.008), open surgery (*P* = 0.032), no PCIA application (*P* = 0.021), longer surgery duration (*P* < 0.001), higher postoperative ANC (*P* = 0.044), higher postoperative ALC (*P* = 0.010), and higher postoperative NLR (*P* = 0.004). Further analysis of the changed ratio showed that a higher changed ratio of WBC count, ANC, and NLR significantly correlated with CPSP (**Figure 2**).

**Table 2:** Main characteristics of patients with or without CPSP after surgery.

Characteristics	CPSP (n=131)	No CPSP (n=837)	*P* value
Age (year)	51 ± 12	47 ± 13	**0.008**
Sex (male/female)	32/99 (24.4/75.6%)	248/589 (29.6/70.4%)	0.222
Height (cm)	159.34 ± 7.31	160.57 ± 8.06	0.808
Weight (kg)	59.73 ± 10.38	61.46 ± 10.89	0.543
BMI (kg/m^2^)	23.50 ± 3.56	23.88 ± 5.27	0.871
Surgical approach (endoscope/open)	111/20 (84.7/15.3%)	760/77 (90.8/9.2%)	**0.032**
ASA (II/III)	53/78 (40.5/59.5%)	496/341 (59.3/40.7%)	**<0.001**
Maximum incisional NRS score at 24h post operation	3.88 ± 1.55	3.36 ± 1.36	0.261
Additional analgesia (Yes/No)	47/84 (35.9/64.1%)	251/586 (30.0/70.0%)	0.174
PCIA (Yes/No)	97/34 (74/26%)	533/304 (63.7/36.3%)	**0.021**
TAP (unilateral/bilateral)	44/87 (33.6/66.4%)	328/509 (39.2/60.8%)	0.220
Type of surgery (gastrointestinal/hepatobiliary/gynecological)	32/53/46 (24.4/40.5/35.1%)	191/342/304 (22.8/40.9/36.3%)	0.915
Surgery duration (min)	150 [80, 235]	105 [65, 175]	**<0.001**
Preoperative WBC	5.49 [4.51, 6.85]	5.89 [4.85, 6.98]	0.126
Preoperative ANC	3.30 [2.60, 4.54]	3.64 [2.90, 4.74]	0.061
Preoperative ALC	1.47 [1.12, 1.84]	1.54 [1.18, 1.88]	0.808
Preoperative NLR	2.19 [1.60, 3.48]	2.39 [1.75, 3.48]	0.253
Postoperative WBC	10.30 [7.66, 13.37]	9.94 [7.52, 12.35]	0.110
Postoperative ANC	8.87 [5.93, 11.43]	8.04 [5.78, 10.33]	**0.044**
Postoperative ALC	1.06 [0.80, 1.16]	1.20 [0.90, 1.62]	**0.010**
Postoperative NLR	7.70 [5.02, 12.11]	6.61 [4.05, 10.06]	**0.004**

CPSP indicates chronic postsurgical pain; BMI indicates body max index; ASA indicates American Society of Anesthesiologists physical status; PCIA indicates Patient Controlled Intravenous Analgesia; TAP indicates transversus abdominis plane block; WBC indicates white blood cell count; ANC indicates absolute neutrophil count; ALC indicates absolute lymphocyte count; NLR indicates neutrophil-lymphocyte ratio.

Continuous data were summarized as the mean ± SD or median (25th–75th percentile), qualitative data were summarized as the number of subjects and percentage. All percentages in parentheses refer to the column group.

Bold values are statistically significant (P < 0.05).

Based on statistical significance or possible clinical implications, variables with *P* < 0.2 in the univariate analysis were included in the multivariate logistic regression model. As shown in **Table 3**, three risk factors were identified for CPSP: surgery duration (OR 1.004, 95% CI, 1.002–1.006; *P* < 0.001), preoperative ANC (OR 1.053, 95% CI 1.002–1.107, *P* = 0.044), and changed ratio of NLR (OR 1.068, 95% CI 1.016–1.124, *P* = 0.011) (**Table 3**). Within both the univariate and multivariate models, the variable of changed NLR ratio had a significant positive correlation with CPSP. As expected, patients with a longer surgery duration were more likely to develop CPSP.

**Table 3:** Multivariate model for CPSP.

Variables	Wald *x^2^*	OR (95% CI)	*P* value
Surgery duration	15.74	1.004 (1.002, 1.006)	**< 0.001**
Preoperative ANC	4.08	1.053 (1.002, 1.107)	**0.044**
Changed ratio of NLR	6.53	1.068 (1.016, 1.124)	**0.011**

ANC indicates absolute neutrophil count; ALC indicates absolute lymphocyte count; NLR indicates neutrophil-lymphocyte ratio.

Bold values are statistically significant (*P* < 0.05).

### 3.3 Sensitivity and subgroup analyses of changes in NLR cutoff at 5 for risk stratification

**Figure 3:**
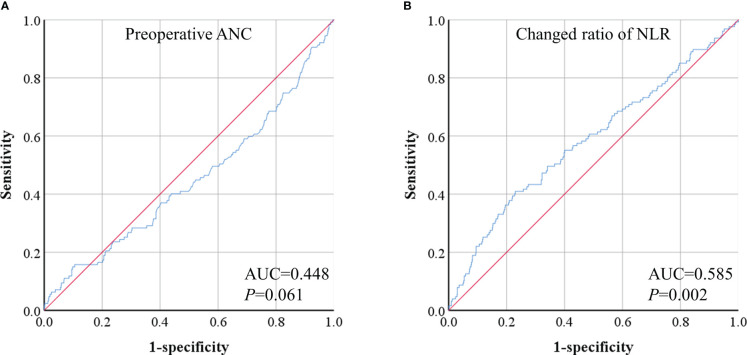
ROC analyses of preoperative absolute neutrophil count (ANC) **(A)** and changed ratio of neutrophil-lymphocyte ratio (NLR) **(B)**.

The predictive model for CPSP yielded an area under the receiver operating characteristic curve of 0.585 (**Figure 3**), and the model showed good calibration with *P* = 0.002.

**Figure 4:**
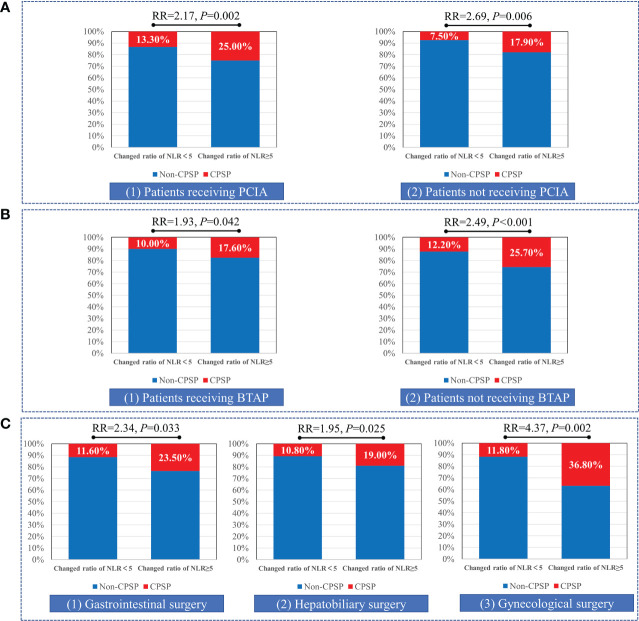
Subgroup analyses of changed ratio of NLR cutoff at 5 for risk stratification. Patients were divided into change ratio of NLR≥5 and change ratio of NLR <5 groups in the subgroup analyses of patient-controlled intravenous analgesia (PCIA) application **(A)**, bilateral transversus abdominis plane (BTAP) block **(B)**, and three different surgery types **(C)**.

Patients were divided into the changed ratio of NLR ≥ 5 and changed ratio of NLR < 5 groups according to the cutoff value at 5. A higher prevalence of CPSP (*P* < 0.001), moderate to severe pain (*P* = 0.009), maximum NRS score after hospital discharge (*P* < 0.001), and overall impact on sleeping, daily life, or work (*P* = 0.030) were associated with a changed ratio of NLR ≥ 5 (**Table 4**). In subgroup analyses, changes in NLR ≥ 5 were associated with CPSP in both patients receiving PCIA (RR = 2.17, *P* = 0.002) and patients not receiving PCIA (RR = 2.69, *P* = 0.006); both patients receiving BTAP (RR = 1.93, *P* = 0.042) and patients not receiving BTAP (RR = 2.49, *P* < 0.001); and all gastrointestinal surgery (RR = 2.34, *P* = 0.033), hepatobiliary surgery (RR = 1.95, *P* = 0.025), and gynecological surgery (RR = 4.37, *P* = 0.002) (**Figure 4**).

**Table 4:** The extent of CPSP and its impact on life of patients with changed ratio of NLR ≥5 or <5 in the perioperative period.

Characteristics	changed ratio of NLR ≥ 5 (n=196)	changed ratio of NLR < 5 (n=772)	*P* value
CPSP (with/without)	43/153 (21.9/78.1%)	88/684 (11.4/88.6%)	**< 0.001**
Overall impact (yes/no)	18/178 (9.2/90.8%)	40/732 (5.2/94.8%)	**0.030**
Maximum NRS score after hospital discharge	1 [0, 3]	0 [0, 2]	**< 0.001**
Moderate to severe pain (with/without)	25/171 (12.8/87.2%)	54/718 (7.0/93.0%)	**0.009**

CPSP indicates chronic postsurgical pain; NRS indicates numeric rating scale; Overall impact indicates interferes with any one of the three: sleep, life, and work; NLR indicates neutrophil-lymphocyte ratio.

Continuous data were summarized as the mean ± SD or median (25th–75th percentile), qualitative data were summarized as the number of subjects and percentage. All percentages in parentheses refer to column group.

Bold values are statistically significant (*P* < 0.05).

## 4 Discussion

In the present study, we investigated the incidence of CPSP at the time after abdominal surgery (11–13 months postoperatively) in 968 patients (13.53%). Furthermore, our study showed that patients with CPSP were older and had a higher ASA grade, longer surgery duration, and higher postoperative plasma inflammatory blood cell parameters (ANC, ALC, and NLR). Open surgery and PCIA were found to be significant independent risk factors for chronic pain development after abdominal surgery. The results of the multivariate logistic regression analysis showed that longer surgery duration, higher preoperative ANC, and higher changed ratio of NLR were risk factors for CPSP. A model for the prediction of CPSP within 24 h after surgery was developed. The transition of acute pain into CPSP could be predicted 24 h after surgery by the model, including surgery duration, preoperative ANC, and the changed ratio of NLR. The most reliable predictive indicator was whether a changed ratio of NLR ≥ 5 is recommended for clinical practice.

Previously published data indicated that the incidence of CPSP after abdominal surgery was 17–40%, which may be attributed to the lack of standardized criteria to classify subjects with CPSP, variation in follow-up periods, diverse surgical procedures, and diagnostic tools to assess CPSP (20, 26–30). In the present study, the prevalence of CPSP after gastrointestinal, hepatobiliary, and gynecological surgeries was 14.35%, 13.42%, and 13.14%, respectively, which is lower than the range previously reported. Possible reasons could be the different interview times for CPSP after abdominal surgery; 11–13 months in this study vs. 4–18 months in other studies (20, 26), different surgical approaches including endoscope and open surgery in this study vs. only open in some reported studies (30), and different postoperative pain management including unilateral or bilateral TAP, with or without PCIA, and additional analgesia in this study vs. no epidural analgesia in some other studies (26).

This study suggests that the changed ratio of NLR can be a potential predictive biomarker for CPSP and quality of life. Postoperative plasma levels of inflammatory cytokines (IL-10, IL-1β, vascular endothelial growth factor, and IL12/IL23p40) were found to be significantly different in patients who developed persistent postoperative pain (31). Compared to plasma inflammatory cytokines, immune cell indicators are more commonly used and readily available in the clinical perioperative period, as surgical patients require routine preoperative and postoperative blood counts. Our study indicated that a higher postoperative ANC, lower postoperative ALC, and higher postoperative NLR were associated with a high incidence of CPSP. Further analysis revealed that the changed WBC ratio, ANC, and NLR significantly correlated with CPSP, and a changed NLR ratio greater than 5 was a good predictor of CPSP in different procedure types, whether the patient received PCIA or unilateral or bilateral TAP. Whether CPSP affects the quality of life is an important indicator of pain intensity and the benefits of intervention assessment (32). Our data suggest that the changed NLR ratio can be a good predictor of CPSP and its impact on quality of life. This indicates good clinical and social significance.

As a reliable and readily available marker of immune response to various infectious and non-infectious stimuli, NLR is widely used across various medical disciplines, such as cancer, atherosclerosis, infection, inflammation, psychiatric disorders, and stress (25, 33–37). Some studies have also investigated the association between NLR and acute postoperative pain (38, 39), but few studies have addressed the role of NLR in CPSP. Neutrophils are among the first-line defenses in inflammatory states, and reactive oxygen species, myeloperoxidase, and proteolytic enzymes are released to destroy pathogens or damaged cells when neutrophils are activated (40). Lymphocytes are also involved in the regulation of the inflammatory response. The number of lymphocytes decreases during acute stress periods, such as surgery, but a long-term decrease in lymphocyte numbers may lead to poor clinical outcomes (41). The NLR is an integrated marker of two inflammatory components and can, therefore, effectively convey the balance between neutrophils and lymphocytes in complex inflammatory activities (42). Therefore, the changed NLR ratio can remarkably reflect the difference in preoperative and postoperative immune status, which was associated with the incidence of CPSP in our study. Some meta-analyses have shown that treatment with nonsteroidal anti-inflammatory drugs can significantly reduce the prevalence of CPSP (43), which is in line with our findings. The mechanisms underlying the effects of this change in perioperative inflammatory status on CPSP remain to be explored. Since the degree and duration of acute postoperative pain are independent risk factors for CPSP (4), the altered perioperative inflammatory status may influence CPSP by affecting acute postoperative pain. NRL is also associated with perioperative stress status (37), whereas perioperative stress is an independent risk factor for CPSP (44); therefore, NRL may interact with stress and affect CPSP. However, further studies are required to explore the underlying mechanisms involved.

Age, sex, and BMI are controversial risk factors for CPSP. Younger age, female sex, and higher BMI are usually considered to be associated with an increased risk of developing CPSP (4, 45–48), but some studies have reached different conclusions (10, 49–51). However, female sex and higher BMI were not independent risk factors for CPSP, and CPSP was more apparent in older age in this study. Our univariate analysis confirmed the results of previous studies that open surgery, higher ASA score, and longer surgery duration are risk factors for CPSP (4, 46).

The limitations of this study need to be addressed as follows: this study was exclusively based on patients receiving surgery at one university-affiliated hospital; the possibility of findings in other hospitals was not assessed; the prediction model was validated in the subgroup analysis of this two-way cohort, but has not been validated in other independent datasets; the type of surgery only included abdominal surgeries such as gastrointestinal, hepatobiliary, and gynecological surgery; other surgeries may have come to different results; the follow-up data came from telephone interviews, so the reliability of this study is strictly dependent on the memory of the patients; out-of-hospital analgesic treatment after hospital discharge was not investigated due to the possibility of high heterogeneity of outcomes; and whether the CPSP was a continuation of already existing preoperative pain was not ruled out.

In summary, this study showed that 13.53% of patients who underwent abdominal surgery developed CPSP. Older age, higher ASA grade, longer duration of surgery, open surgery, PCIA application, and higher postoperative plasma inflammatory blood cell parameters (ANC, ALC, and NLR) could be associated with CPSP. The changed ratio of NLR could be used for the early identification of patients at risk for CPSP to alert clinicians to undertake further assessment.

## Data availability statement

The raw data supporting the conclusions of this article will be made available by the authors, without undue reservation.

## Ethics statement

The studies involving human participants were reviewed and approved by The Medical Ethics Committee of the Second Affiliated Hospital of Chongqing Medical University. Written informed consent for participation was not required for this study in accordance with the national legislation and the institutional requirements.

## Author contributions

BS, FX, XZ, YZ, QL, SL, JC, and YC contributed to the data collection. BS and GD contributed to data analysis. BS, GD, and HH contributed to the draft writing. GD and HH contributed to draft revision. All authors gave final approval for the version to be published and agreed to be accountable for all aspects of the work.
